# A Subunit Vaccine Based on the VP2 Protein of Porcine Parvovirus 1 Induces a Strong Protective Effect in Pregnant Gilts

**DOI:** 10.3390/vaccines11111692

**Published:** 2023-11-05

**Authors:** Zhanye Ling, Huawei Zhang, Yingjin Chen, Leqiang Sun, Junlong Zhao

**Affiliations:** 1 State Key Laboratory of Agricultural Microbiology, Huazhong Agricultural University, Wuhan 430070, China; zhanyeling@mail.hzau.edu.cn (Z.L.); zhw@mail.hzau.edu.cn (H.Z.); cyj23@mail.hzau.edu.cn (Y.C.); 2 College of Veterinary Medicine, Huazhong Agricultural University, Wuhan 430070, China; 3 Xinxin Livestock Co., Ltd., Huang-Fan Qu, Zhoukou 466600, China

**Keywords:** porcine parvovirus 1, VP2 protein, subunit vaccine, protective immunity

## Abstract

Porcine parvovirus 1 (PPV1) is one of the most prevalent pathogens that can cause reproductive disorder in sows. The VP2 protein of PPV1 is the most important immunogenic protein that induces neutralizing antibodies and protective immunity. Thus, VP2 is considered an ideal target antigen for the development of a genetically engineered PPV1 vaccine. In this study, the baculovirus transfer vector carrying the HR5-P10-VP2 expression cassette was successfully constructed with the aim of increasing the expression levels of the VP2 protein. The VP2 protein was confirmed using SDS‒PAGE and Western blot analyses. Electronic microscope analysis showed that the recombinant VP2 proteins were capable of self-assembling into VLPs with a diameter of approximately 25 nm. The immunogenicity of the VP2 subunit vaccine was evaluated in pigs. The results showed that VP2 protein emulsified with ISA 201VG adjuvant induced higher levels of HI antibodies and neutralizing antibodies than VP2 protein emulsified with IMS 1313VG adjuvant. Furthermore, the gilts immunized with the ISA 201VG 20 μg subunit vaccine acquired complete protection against PPV1 HN2019 infection. In contrast, the commercial inactivated vaccine provided incomplete protection in gilts. Therefore, the VP2 subunit vaccine is a promising genetically engineered vaccine for the prevention and control of PPV1.

## 1. Introduction

Porcine parvovirus 1 (PPV1) is a small, nonenveloped, single-stranded DNA virus that is approximately 4.8 kb long [1]. It belongs to the genus Protoparvovirus within the family Parvoviridae. PPV1 is the only virus that has been successfully isolated in cell culture, and it is universally acknowledged that this pathogen is associated with reproductive failure [2]. The genome of PPV1 contains two open reading frames, with the left side encoding nonstructural proteins (NS1, NS2, and NS3) and the right side encoding structural proteins (VP1 and VP2) [3,4]. The VP2 protein, which is the major immunogenic protein, elicits protective immunity against PPV1 infection [5]. Additionally, in eukaryotic expression systems, the VP2 protein can be independently assembled into virus-like particles (VLPs). Other novel parvoviruses include PPV2 and PPV3, PPV4–PPV6, and PPV7, which belong to the genera Tetraparvovirus, Copiparvovirus, and Chapparvovirus, respectively [6,7,8,9,10].

PPV1, which is associated with SMEDI syndrome (stillbirth, mummification, embryonic death, infertility), is a virus of which pigs are natural hosts [2,3,11]. Pigs, such as stock boars, gilts, and sows, are particularly prone to PPV1 infection [2,3,11]. The virus causes an acute sow reproduction disorder by infecting the embryo and fetus through vertical transmission via the placenta. PPV1 is a major contagious disease that has been widely reported in pig farms worldwide [12,13,14]. While vaccines are an effective method to prevent PPV1 infection, inactivated vaccines for PPV1 cannot inhibit viremia, leading to immunity failure [5,11,15,16].

In China, the insect-cell-based baculovirus expression vector system (BEVS) is currently one of the primary methods for producing PCV2 VLPs [17,18]. However, under normal conditions, the protein production of BEVS is limited compared with CHO cell expression. This limitation can be addressed by utilizing insect cell lines in BEVS, modifying the baculovirus transfer vector, or utilizing RNA interference technology to provide recombinant products [19,20,21]. An important cis-regulatory element for protein expression in insect cells is the promoter. Previous studies have demonstrated that the combination of the homologous region of the baculovirus with the promoter can significantly enhance the expression of recombinant proteins in insect cells [21,22,23,24].

In this study, a recombinant baculovirus expressing VP2 protein, in conjunction with the p10 promoter and homologous region 5 (HR5), was constructed to enhance VP2 protein expression. The protective immune responses of the VP2 protein were then investigated in pigs. In particular, the VP2 protein emulsified with ISA 201VG was found to induce high levels of humoral immune response. Furthermore, the VP2 protein could confer complete protection against PPV1 infection in pregnant gilts.

## 2. Materials and Methods

### 2.1. Cells and Antibodies

Sf9 cells (ATCC; CRL-1711™) were grown in Sf-900™ II SFM (Gibco, New York, NY, USA). High Five™ cells (B85502) were grown in Express Five™ SFM (Gibco, New York, NY, USA). ST cells (ATCC; CRL-1746) were grown in Dulbecco’s modified essential medium (Gibco, New York, NY, USA) containing 10% fetal bovine serum (Gibco, New York, NY, USA) at 37 °C in a humidified 5% CO_2_ incubator. Alexa Fluor 488 goat anti-mouse was obtained from Life Technologies (Life Technologies Corporation, Carlsbad, CA, USA). Horseradish peroxidase (HRP)-conjugated goat anti-mouse IgG was obtained from Sigma Aldrich (Sigma-Aldrich Corporation, Saint Louis, MO, USA).

### 2.2. Construction of Recombinant Baculoviruses

**Figure 1:**
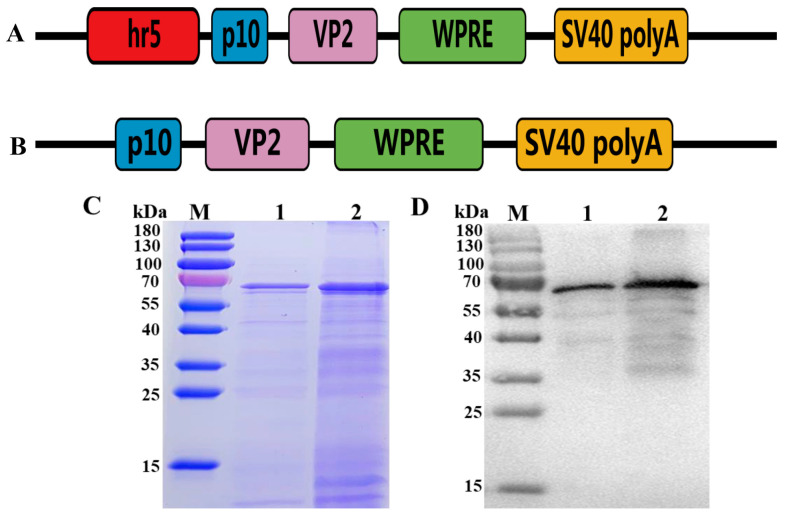
Schematic diagrams of the recombinant transfer vector and identification of recombinant VP2 protein. (**A**) Scheme of pFastBacHT-HR5-P10-VP2. (**B**) Scheme of pFastBacHT-P10-VP2. (**C**) SDS‒PAGE analysis of the recombinant VP2 protein. Lane 1: the culture supernatant of AC-P10-VP2; Lane 2: the culture supernatant of AC-HR5-P10-VP2. (**D**) Western blot analysis of the recombinant VP2 protein. Lane 1: the culture supernatant of AC-P10-VP2; Lane 2: the culture supernatant of AC-HR5-P10-VP2.

The plasmid pUC57-HR5-P10 expression cassette was synthesized (Sangon Biotech, Shanghai, China) and comprised an enhancer element, a P10 promoter, multiple cloning sites (BamHI and HindIII), and a WPRE element. The plasmid pUC57-PPV1-VP2 was synthesized using codon optimization (Sangon Biotech, Shanghai, China). The plasmid pFastBacHT A (Invitrogen, Carlsbad, CA, USA) was used as the backbone of the baculovirus transfer vector. The HR5-P10 expression cassette was subcloned and inserted into the pFastBacHT A vector using a Seamless Cloning and Assembly Kit (NEB, Ipswich, MA, USA). Then, the PPV1-VP2 gene segment was inserted into pFastBacHT-HR5-P10 digested with BamHI and HindIII to obtain the transfer vector pFastBacHT-HR5-P10-VP2 (Figure 1A). The same approach was used to construct the recombinant plasmid pFastBacHT-P10-VP2 (Figure 1B), which did not contain an enhancer element (HR5). Finally, the two recombinant transfer vectors were confirmed by sequencing. Two recombinant baculoviruses (Ac-HR5-P10-VP2 and Ac-P10-VP2) were subsequently generated using the Bac-to-Bac system (Invitrogen) following the manufacturer’s instructions (Invitrogen, Carlsbad, CA, USA).

### 2.3. Detection of VP2 Protein Expression and Purification

High Five™ cells were infected with Ac-HR5-P10-VP2 and Ac-P10-VP2 at a multiplicity of infection (MOI) of 0.005. At 120 h postinfection, the infected cell lysates were determined using the Micro BCA™ Protein Assay Kit (Thermo Scientific, Waltham, MA, USA). The protein samples were then separated through sodium dodecyl sulfate (SDS)–polyacrylamide gel electrophoresis (PAGE) on a 12% polyacrylamide gel and then transferred onto polyvinylidene fluoride (PVDF) membranes (Roche, Basel, Switzerland). The membranes were blocked with 5% nonfat milk in PBST, incubated with anti-VP2 MAb (1:500 dilution; prepared in our laboratory), and incubated again with anti-mouse IgG antibodies conjugated to horseradish peroxidase (1:4000 dilution, Sigma, Saint Louis, MO, USA). Protein bands were detected using the ECL Chemiluminescence Detection Kit and Image Lab 4.0 software (Bio-Rad, Shanghai, China).

For the purification of the VP2 protein, High Five™ cells were infected with Ac-HR5-P10-VP2 at an MOI of 0.005. After incubation for 7–8 days, the culture supernatants were centrifuged at 12,000 rpm for 30 min. The VP2 protein was then purified using anion exchange chromatography (GE, Chicago, IL, USA) following the manufacturer’s instructions.

### 2.4. Transmission Electron Microscopy (TEM)

High Five™ cells were infected with Ac-HR5-P10-VP2 at an MOI of 0.005. After incubation for 7–8 days, the supernatant was centrifuged at 13,400× *g* for 30min at 4 °C. Subsequently, the supernatant was centrifuged at 125,000× *g* for 4 h with a Beckman SW28 rotor by using a 35% sucrose cushion. Protein samples were harvested for electron microscopy analysis by using an H-7000FA electron microscope (Hitachi Co., Tokyo, Japan).

### 2.5. Analysis of Immunogenicity

Different kinds of immune adjuvants and antigen concentrations were designed to assess the immunogenicity of the VP2 protein. Six-week-old, crossbred weaning piglets were randomly divided into six groups. Each group was composed of five piglets. The piglets were confirmed to be seronegative for PPV1 by using a hemagglutination inhibition (HI) assay. All experimental protocols were approved and performed by the Laboratory Animal Ethical and Welfare Committee of Hubei Province, China (Approval No. 20233145).

Ten micrograms of purified VP2 protein was emulsified with the ISA 201VG adjuvant (Seppic, Paris, France) at a ratio of 1:1 (*w*/*o*/*w*) or the IMS 1313VG adjuvant (Seppic, Paris, France) at a ratio of 1:1 (*w*/*w*) in accordance with the manufacturer’s instructions. Twenty micrograms of purified VP2 protein was emulsified with the ISA 201VG adjuvant or the IMS 1313VG adjuvant in the same manner. Pigs in groups A and B were injected intramuscularly with 2 mL of ISA 201VG 10 μg and ISA 201VG 20 μg VP2 subunit vaccines, respectively. Pigs in groups C and D were injected intramuscularly with 2 mL of IMS 1313VG 10 μg and IMS 1313VG 20 μg VP2 subunit vaccines, respectively. Group E, the positive control group, was immunized with 2mL of the commercial inactivated vaccine (Tianjin Ringpu Biotechnology Co., Ltd., Tianjin, China). Group F, the negative control group, was immunized with 2mL of PBS. Blood samples were collected at 0, 21, 35, 60, and 90 days postimmunization. Serum samples were tested for PPV1-specific neutralizing antibodies and HI antibody titer.

### 2.6. HI Assay

Fifty microliters of serum samples were serially diluted twofold and mixed with equal volumes of 4 HA units of the PPV1 HN19 strain and incubated at 37 °C for 60 min. Then, 50 microliters of 1% guinea pig red blood cell suspension was added and incubated at 37 °C for 45 min. The positive and negative controls were included in the experiment. The HI antibody titer was counted as the highest serum dilution at which erythrocyte aggregation was suppressed completely [5].

### 2.7. Neutralization Test

The neutralization test was performed as described previously [5,25]. The serum samples were heat inactivated at 56 °C for 30 min. Then, 50 microliters of serum samples were serially diluted twofold and mixed with 200 TCID_50_ PPV1 HN19 strains at 37 °C for 60 min. Serum–virus mixtures were added to confluent ST cells cultured in 96-well plates and then incubated at 37 °C for 4 days. The cells were observed for cytopathic effect (CPE). The neutralizing antibody titer was counted as the reciprocal of the highest serum dilution at which no CPE was observed.

### 2.8. Challenge Experiment

Twelve five-month-old PPV-negative gilts were purchased from the experimental farm of Huazhong Agricultural University and randomly divided into three groups with four gilts per group. Group A was injected intramuscularly with 2 mL of the ISA 201VG 20 μg VP2 subunit vaccine. Group B was injected intramuscularly with 2 mL of the commercial inactivated vaccine (Tianjin Ringpu Biotechnology Co., Ltd., Tianjin, China). Group C, the negative control group, was immunized with 2 mL of PBS. One month after immunization, all pigs were artificially inseminated as many times as required with PPV-free sperm in two or three days. At approximately day 40 of pregnancy, all gilts were challenged with 2 mL of the virulent PPV1 HN19 strain intranasally and 2 mL intramuscularly (10^6.0^TCID_50_/mL). The gilts were checked for clinical signs (abortion, mummification, and death) and were recorded until the piglets were born. Blood samples were collected at 0, 14, and 28 days postimmunization and at 0, 7, 14, 21, 35, and 60 days postchallenge (dpc). The serum samples were tested for HI antibody titers. EDTA blood and nasal swab samples were collected at 0, 3, 7, 14, 21, 35, and 60 dpc for detecting viremia and monitoring virus excretion. Viral DNA was extracted using a DNA extraction kit (Code No. 9766, TaKaRa, Dalian, China). A real-time fluorescent quantitative PCR test was performed to detect PPV1 as described previously [9]. The real-time PCR was carried out in the ABI ViiA7 system (Thermo Fisher, Waltham, WA, USA). Two primers (PPV1F: 5′-CAGAATCAGCAACCTCACCA-3′ and PPV1R: 5′-GCTGCTGGTGTGTATGGAAG-3′) and one probe (FAM-5′-TGCAAGCTTAATGGTCGCACTAGACA-3′-TAMRA) were used for amplification. The real-time PCR reactions were performed in a 20 μL final volume, containing 1 μL of the cDNA template, 400 nM of each primer, and 200 nM of each probe. The amplification was performed under the following conditions: initial denaturation at 95 °C for 5 min, 40 cycles of denaturation at 95 °C for 15 s, annealing at 58 °C for 30 s, elongation at 72 °C for 30 s, and a final extension step at 72 °C for 5 min. The testing samples with Ct values ≥ 37 were considered negative. All experimental protocols were approved and performed by the Laboratory Animal Ethical and Welfare Committee of Hubei Province, China (Approval No. 20230617).

### 2.9. Statistical Analysis

Statistical analysis was performed using GraphPad Prism (GraphPad Software7.0, San Diego, CA, USA). All data were analyzed with one-way analysis of variance using the GraphPad Prism software (Normality and Lognormality Tests). *p* < 0.05 was considered to be statistically significantly different.

## 3. Results

### 3.1. Expression of VP2 Protein

Ac-HR5-P10-VP2 and Ac-P10-VP2 were created in Sf9 cells by using the Bac-to-Bac system. The supernatant of infected High Five™ cells was harvested and detected by experiment after observing that the CPE reached eighty percent or greater. SDS‒PAGE and Western blot results displayed a protein band at approximately 64 kDa (Figure 1), indicating protein expression. Interestingly, the expression of VP2 in Ac-P10-VP2 was found to be lower than that in Ac-HR5-P10-VP2, despite both proteins being loaded at the same quantity in the protein sample. Furthermore, the VP2 protein was purified using an anion exchange chromatography method. The AKTA Protein Purification System revealed that the recombinant VP2 protein eluted with buffer B (20 mmol/L Tris + 500 mmol/L NaCl, pH 7.0) (Supplementary Figure S1).

### 3.2. Electron Microscopy

**Figure 2:**
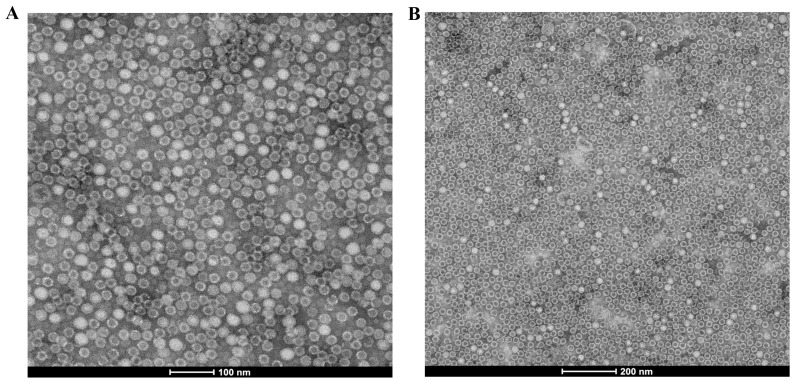
Analysis of the recombinant VP2 proteins through electron microscopy. The recombinant VP2 proteins were prepared by ultracentrifugation and negatively stained with 2% (wt/vol) aqueous uranyl acetate. (**A**) Scale bar indicates 100 nm. (**B**) Scale bar indicates 200 nm.

TEM assays were performed to verify whether the VP2 protein expressed in High Five™ cells could self-assemble into VLPs. The recombinant VP2 protein could form VLPs with a diameter of approximately 25 nm, as shown in Figure 2. These VLPs were morphologically similar to mature PPV1 virions.

### 3.3. Immunogenicity Study

**Figure 3:**
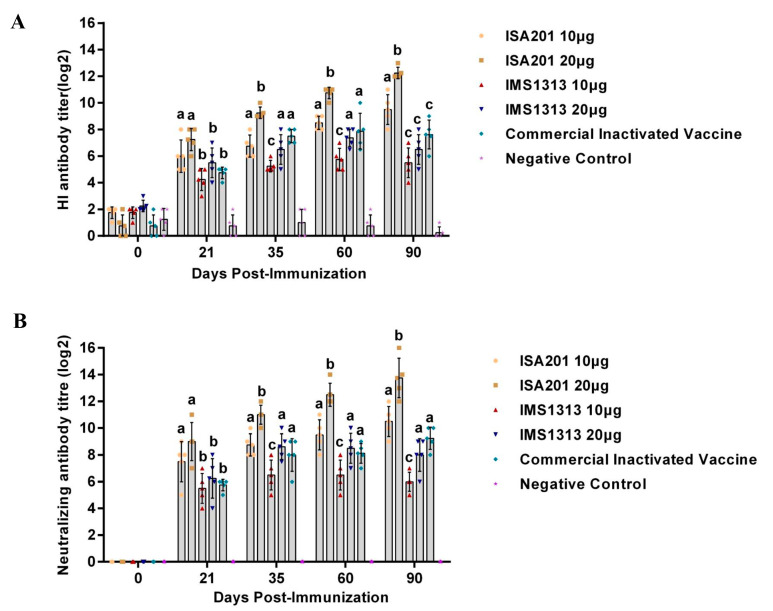
Detection of PPV1-specific antibody in the sera of the immunized pigs. (**A**) Serum HI antibody titers and (**B**) neutralization antibody titers in different groups. The sample size was five piglets. All data are expressed as the mean ± SEM. The data were analyzed by using one-way ANOVA to compare the difference among groups immunized with different vaccine at the same time. Different letters (a and b, c) indicate a statistically significant difference between different groups (*p* < 0.05).

The different antigen contents of the VP2 protein were evaluated using various immune adjuvants. At 21 dpi, the ISA 201VG 20μg and ISA 201VG 10 μg groups exhibited significantly higher PPV1 HI antibody titers than the other groups (Figure 3A). The HI antibody titers induced by different vaccines varied over time. Notably, the pigs vaccinated with ISA 201VG 20 μg demonstrated consistently higher HI antibody titers compared with all other groups.

The serum samples were also analyzed for their ability to neutralize PPV1 using a neutralization test. At 21 dpi, the ISA 201VG 20 μg and ISA 201VG 10 μg groups showed significantly higher neutralizing titers against PPV1 than the other groups (Figure 3B). At 35, 60, and 90 dpi, the differences in neutralizing titers between the ISA 201VG 10 μg, IMS1313VG 20 μg, and commercial inactivated vaccine groups were not significant. However, the mean neutralization titers induced by ISA 201VG 20 μg were higher than those of any other group.

As the antigen concentration increased, both the HI antibody titer and the neutralizing antibody titer steadily increased during the course of immunization. Notably, ISA 201VG demonstrated a significantly higher immune effect than IMS 1313VG when administered at the same dose. Furthermore, the trial results confirmed that pigs immunized with the ISA 201VG 20 μg VP2 subunit vaccine elicited high-level immune responses.

### 3.4. Protection of Pregnant Gilts against PPV1 Challenge

**Figure 4:**
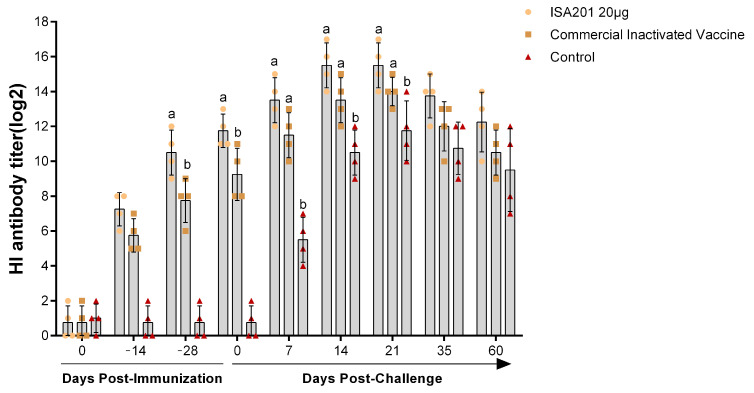
Detection of HI antibody titers in gilts after vaccination and PPV1 challenge. The sample size was four gilts. All data are expressed as the mean ± SEM. The data were analyzed by using one-way ANOVA to compare the difference among groups immunized with different vaccine at the same time. Different letters (a, and b) indicate a statistically significant difference between different groups (*p* < 0.05).

The ISA 201VG 20 μg subunit vaccine and the commercial inactivated vaccine were administered as a single vaccination to evaluate their protective efficacy in pregnant gilts. As shown in Figure 4, all gilts vaccinated with ISA 201VG 20 μg or the commercial inactivated vaccine developed specific PPV1 HI antibodies as early as 14 dpi. The average antibody titers (HI titers) were 1:152.2 and 1:53.8, respectively (Figure 4). At 28 dpi, the differences between the ISA 201VG 20 μg and commercial inactivated vaccine groups were significant. At 7 dpc, the negative control group was detected, and the sera samples were HI antibody positive. The serum antibody titers of all three groups peaked at 21 dpc, with mean antibody titers of 1:46341, 1:16384, and 1:3444 (Figure 4).

**Figure 5:**
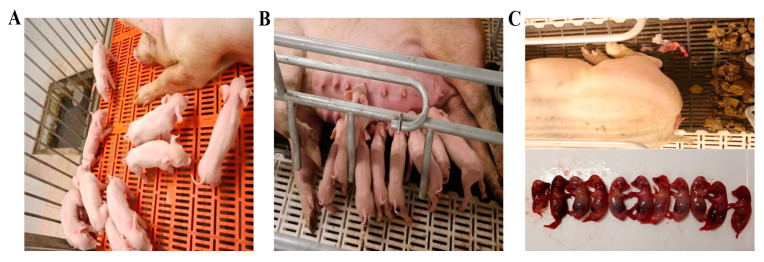
Observation of clinical symptoms in gilts after PPV1 challenge. (**A**) ISA 201VG 20 μg VP2 subunit vaccine group. (**B**) Commercial inactivated vaccine. (**C**) Negative control group.

All gilts vaccinated with ISA 201VG 20 μg or the commercial inactivated vaccine were observed and showed no clinical signs before and after challenge. However, four pregnant gilts in the negative control group had a miscarriage or stillbirth (Figure 5). As shown in Table 1, the fetuses of all pregnant gilts in the ISA 201VG 20 μg and commercial inactivated vaccine groups survived, while the pregnant gilts in the negative control group had 42 fetuses, of which 3 were alive, 8 were dead, and 31 were mummified.

**Figure 6:**
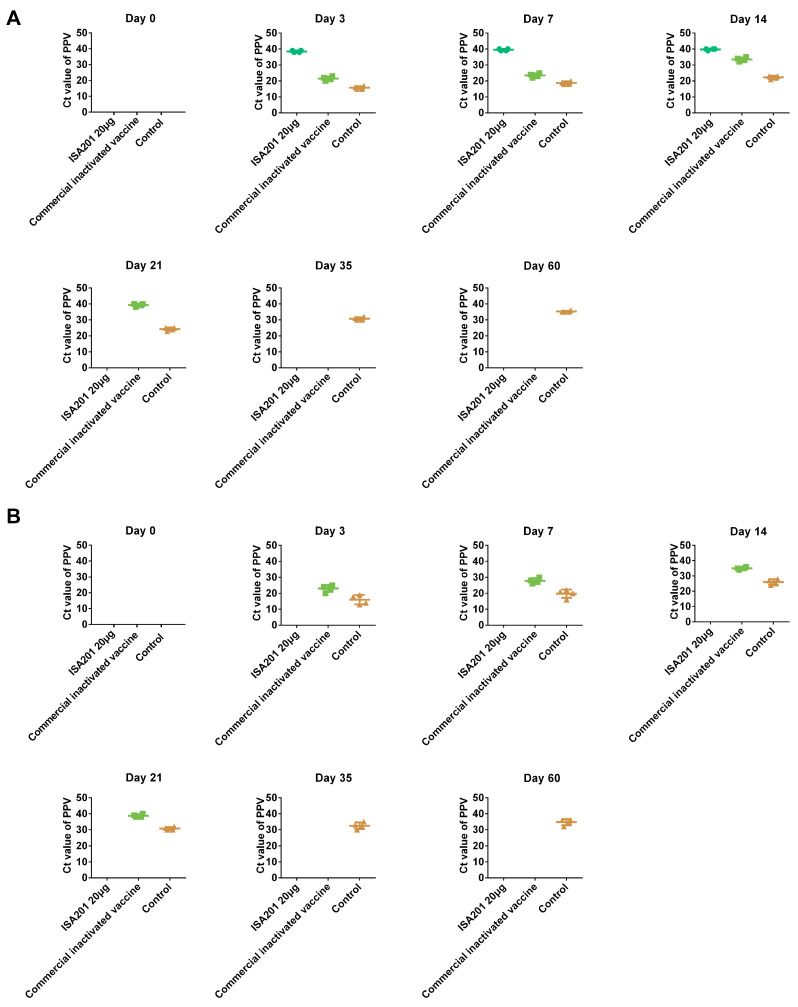
Detection of PPV1 DNA in the nasal swab (**A**) and blood (**B**) samples after challenge by real-time RT‒PCR. The samples were collected at 0, 3, 7, 14, 21, 35, and 60 dpc, and DNA was extracted using a DNA isolation kit (TIANGEN, Beijing, China). The virus content was determined using real-time RT‒PCR as described previously; the samples with Ct values of ≥37 were considered negative or undetectable; the samples with Ct values < 37 were considered positive. The missing panels indicated that the Ct number was not detected.

Before vaccination, all gilts tested negative for PPV1 under real-time RT‒PCR. Nasal swab and blood samples were collected at 0, 3, 7, 14, 21, 35, and 60 dpc, and PPV1 DNA was detected using real-time RT‒PCR. After the challenge, PPV1 DNA was detected in all gilts in the control group. However, no PPV1 DNA was detected in any of the gilts immunized with the ISA 201VG 20 μg subunit vaccine, as shown in Figure 6. Conversely, all gilts immunized with the commercial inactivated vaccine tested positive for PPV1 at 3, 7, and 14 dpc. These results suggest that the commercial inactivated vaccine is ineffective in preventing viremia and excretion of PPV1.

## 4. Discussion

The commercial inactivated vaccine, which includes nine strains of PPV1 belonging to the same branch of the NADL-2 strain, is widely used in China and plays a critical role in controlling the PPV1 epidemic [26]. The incidence of this disease has dropped considerably in the past ten years, with an incidence of 1–2.6% in parts of China. However, immunization failure in large-scale pig farms can still occur owing to the presence of the basic variant of PPV1. Previous studies have shown that PPV1 isolates in China are located in different evolutionary lines [26,27].

A mummified fetus collected from a breeding farm in Nanyang, Henan Province, in March 2019 tested positive for PPV1. A strain of PPV1 named HN2019 was subsequently isolated from the samples by using ST cells. Genome sequence alignments revealed that the HN2019 isolates shared the highest nucleotide identity (99.5%) with the Korean PPV1 strain (GenBank: MW711831.1), which was closely related to the PPV1 27a strain (GenBank: AY684871.1). As a result, the development of novel vaccines by using epidemic isolates has become increasingly important.

Insect-cell-based BEVSs have been developed into an excellent platform for the large-scale expression of foreign proteins [28]. Its advantages include the ability to perform many posttranslational modifications, such as phosphorylation and glycosylation. The BEVS also boasts extremely high expression of foreign genes. Notably, it differs from bacterial systems in that it does not produce bacterial endotoxin, which can lead to severe adverse reactions. As a result, the BEVS has been widely used for the production of VLP vaccines. VLP vaccines have the potential to be a safe and effective alternative to traditional inactivated vaccines because they do not contain viral nucleic acids and their three-dimensional conformation is similar to that of the native virus.

Research has shown that gene expression in eukaryotes is primarily regulated at the transcription level. Previous studies have indicated that HR5, in combination with baculovirus promoters, can considerably enhance gene transcription efficiency and improve the production of recombinant proteins [21,24]. This study successfully constructed a baculovirus transfer vector carrying the HR5-P10-VP2 expression cassette with the aim of increasing the expression levels of the VP2 protein. Evaluation by SDS‒PAGE and Western blot analyses demonstrated that the VP2 protein was efficiently expressed in insect cells. These results confirmed that the VP2 expression of Ac-HR5-P10-VP2 was higher than that of Ac-P10-VP2 under the same conditions. Moreover, electron microscopy analysis further supported previous findings, as it revealed that the recombinant VP2 proteins were capable of self-assembling into VLPs with a diameter of approximately 25 nm.

The immunogenicity of the VP2 protein was evaluated through animal experiments. The results indicated that pigs immunized with the ISA 201VG adjuvant had higher levels of HI antibodies and neutralizing antibodies than those immunized with the IMS 1313VG adjuvant. This finding suggests that the ISA 201VG adjuvant significantly enhances the immune response to the VP2 protein. In general, the levels of HI antibodies detected in pigs vaccinated with the VP2 subunit vaccine matched the levels of neutralizing antibodies. After the PPV1 HN2019 challenge, all pregnant gilts in the negative control group exhibited typical clinical symptoms, such as miscarriage or stillbirth. However, no clinical signs could be observed in the gilts vaccinated with ISA 201VG 20 μg or the commercial inactivated vaccine before and after challenge. Nevertheless, in the early days of challenge, PPV1 DNA was detected in the blood and nasal swab samples from the commercial inactivated vaccine group, which is consistent with previous studies and indicates that this vaccine cannot fully prevent viremia and excretion of PPV1. Meanwhile, no PPV1 DNA was detected in gilts immunized with the ISA 201VG 20 μg subunit vaccine, indicating that this vaccine provides complete protection against PPV1 HN2019 infection.

In conclusion, this study demonstrated that homologous DNA regions (HR5) in combination with the P10 promoter can enhance the expression of VP2 proteins. This finding highlights the ISA 201VG 20 μg VP2 subunit vaccine as a promising and safe candidate for preventing PPV1 infection. Future research should prioritize investigating its protective efficacy against heterologous virus strains to effectively manage and control this disease.

## Figures and Tables

**Table 1:** Vitality in progeny per sow after the challenge with PPV1.

Group	Sow No.	Vitality in Progeny			
		Dead	Mummified	Alive	Total
ISA201VG 20 µg	A01	0	0	8	8
	A02	0	0	12	12
	A03	0	0	13	13
	A04	0	0	9	9
Commercial inactivated vaccine	B11	0	0	11	11
	B12	0	0	9	9
	B13	0	0	10	10
	B14	0	0	12	12
Control	C21	4	4	2	2
	C22	0	12	0	0
	C23	4	6	1	1
	C24	0	9	0	0

## Data Availability

The datasets used and analyzed during the current study are available from the corresponding author on reasonable request.

## Supplementary Materials

The following supporting information can be downloaded at https://www.mdpi.com/article/10.3390/vaccines11111692/s1. Figure S1: The VP2 protein was purified using the AKTA protein purification apparatus. (A) The UV absorption peak value of the purified recombinant VP2 protein. (B) SDS–PAGE analysis of the purified recombinant VP2 protein.
