# Supplementary material for: A Subunit Vaccine Based on the VP2 Protein of Porcine Parvovirus 1 Induces a Strong Protective Effect in Pregnant Gilts
Source: Vaccines (Basel). 2023 Nov 5;11(11):1692. doi: 10.3390/vaccines11111692 (PMC10675385; doi:10.3390/vaccines11111692)

vaccines

Figure S1. The VP2 protein was purified using the AKTA protein purification apparatus. (A) The UV absorption peak value of the purified recombinant VP2 protein. (B) SDS–PAGE analysis of the purified recombinant VP2 protein.

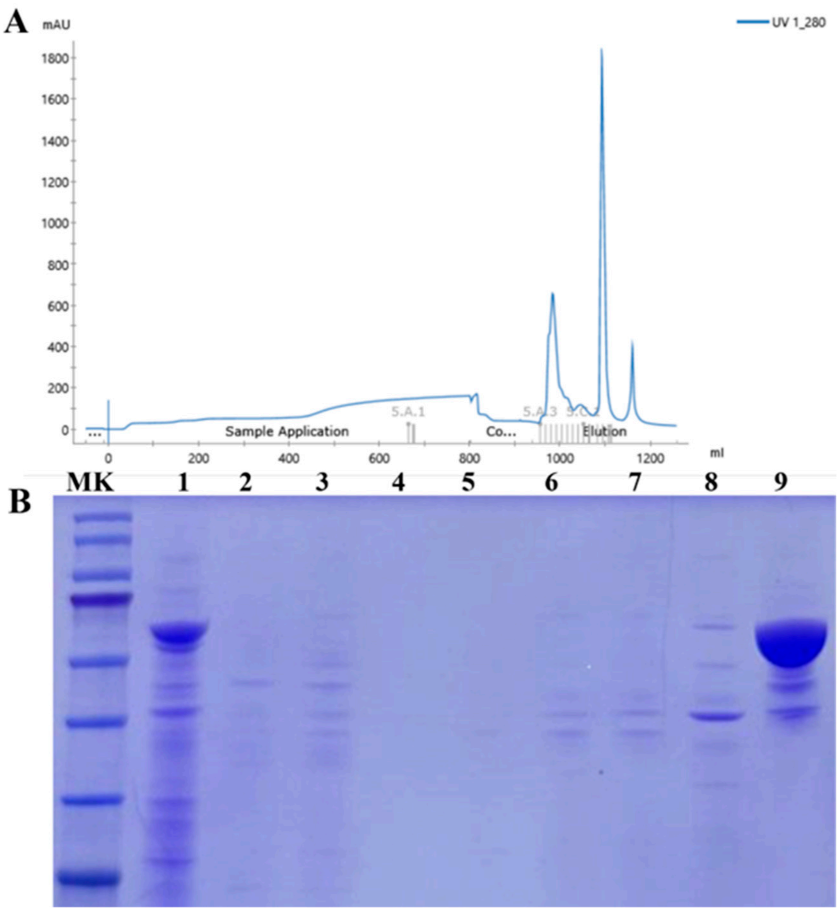

Supplement: Supplementary file 1 [file vaccines-11-01692-s001.zip › vaccines-2621332-supplementary.pdf]
